# Demonstrative Midwifery

**Published:** 1850-03

**Authors:** 


*Demonstrative Midwifery.*—The following letter has been handed to us, with a request that it be inserted in this Journal:—

To Doct. Austin Flint, Editor, &c.

*Sir,*—The undersigned, members of the Medical Profession, have noticed with regret, in the February number of your Journal, the Editorial article, and the correspondence to which it refers, entitled "Demonstrative Midwifery."

The propriety of the exhibition with the living subject, before the graduating class at the College, as we understand it, does not, in our view, admit of a public discussion ; and our only object in this communication is to say, that the practice does not "commend itself to the cordial approbation of the medical profession" of Buffalo, but, on the contrary merits a severe rebuke; because we deem it wholly unnecessary for the purpose of teaching, unprofessional in manner, and grossly offensive, alike to morality, and common decency. For the credit of the medical profession we hope this "innovation" will not be repeated in this, or any civilised community.

Buffalo, Feb. 21st, 1850.

John Hauenstein,

Jno. S. Trowbridge,

E. F. Gray,

J. D. Hill,

H. D. Garvin,

Geo. N. Burwell,

C. C. Wyckoff,

William Ring,

J. Trowbridge,

B. Burwell,

M. Bristol,

A. S. Sprague,

Josiah Barnes,

H. H. Bissell,

Joseph Peabody,

G. F. Pratt,

S. Barrett,

The above expression of opinion and sentiment by seventeen* Physicians of Buffalo, has been called forth (as stated in the communication) by some editorial remarks on the subject of Demonstrative Midwifery, contained in the February number of this Journal. The reader, by referring to those remarks, will perceive, that we then entertained the belief that the plan of illustrating obstetrical instruction with the living subject would commend itself to the cordial approval of the medical profession, as well as others The plan, however, as would seem, does not meet with the cordial approval of those of our medical brethren of this city whose names are attached to the foregoing letter; and, such being the case, they feel it to be a duty to repudiate an imputation which, if they were silent, might rest upon them. This appears to be the posture of the matter, and since our own views are

* The letter, when handed to us, contained the signatures of eighteen Physicians. Since this article was sent to the press, one of the original signers has sent us a note requesting his name to be erased, which has been done. Editor.

in nowise altered, it will be expected that we should submit, in connection with the above communication, some farther remarks on the subject.

We would state, at the outset, that we publish the letter with cheerfulness, and perhaps it is due to ouselves to say that we assented to its insertion in our volumes, even before giving it a perusal. We entertain friendly and respectful feelings toward each of the individuals whose names are appended to it, and we are not aware that these feelings are not reciprocated. We take no exceptions to the paper on the score of personality, nor shall we say aught in reply, dictated in any other spirit than that of kindness. We have said thus much, by way of preface, partly as an apology for devoting to the subject, in the following remarks, greater space than perhaps our readers not residing in this city may think is due to its intrinsic importance.

The point in the letter on which we will first comment, is the statement that the plan of demonstrative midwifery, as practised at the Med. Dept. of the University of Buffalo, does not meet the cordial approbation of the Physicians of Buffalo. In expressing our belief that the plan would meet with the cordial approbation of the medical profession, we did not intend to be understoood to refer exclusively to our medical brethren in this city. We certainly did not anticipate that it would meet with their disapproval, as a body, although we were then aware that it had excited considerable opposition with a few; but by the expression, medical profession, we meant, what the expression conveys, the totality of the profession, not a sectional fraction of it. Our remarks were not addressed solely to those of our Buffalo brethren who do us the honor to peruse our lucubrations, but to all our medical readers. We do not by any means undervalue the character and influence of the medical profession of Buffalo—none can respect it more than we—with the present circulation of our Journal, however, the subscribers who reside in Buffalo constitute but a small numerical portion of its patrons.

But is it clear that the plan does *not* meet with the cordial approbation of the medical profession of Buffalo? It may be that it does not, but the communication which contains the statement that it does not, fails to furnish proof of the correctness of the assertion. There are seventeen names attached to the communication, while there are over forty members of the regular professsion in this city. The opinions or sentiments of a body of individuals are generally supposed to be determined by the rule of plurality. A document signed by the individual names of a minority of the Physicians of Buffalo can hardly be taken as evidence for the statement that the whole profession of the place entertain the views which the document sets forth! Still less can a minority of the Physicians of Buffalo be considered to represent the views of the medical profession of the United States! We do not assume the responsibility of affirming that the majority of the Physicians either of Buffalo, or the United States, will approve of the plan of demonstrative midwifery. We may be mistaken on this point, in so far as our belief is concerned. All we intend to say is, that the communication handed us, in so far as it contains evidence of the approbation of our professional brethren of Buffalo, goes rather to sustain, than disprove the correctness of the opinion advanced by us, since it embraces the names of a minority only of the medical profession of this city.

In reading the letter of our medical friends, our attention was arrested by the language at the commencement of the second paragraph, to wit: "The propriety of the exhibition, with the living subject before the graduating class at the college, *as we understand it*," etc. We have italicised the portion to which we would direct attention. The expression "as we understand it," involves some doubt whether the matter to which it relates was correctly understood. Now whether it has, or has not been correctly understood by all the gentlemen who signed the letter, we do not presume to decide. We have conversed on the subject with but one of the signers, since a committee called upon us to deliver the letter. That individual, we found, had credited a very erroneous statement of it. It may be that all the others were correctly informed. But why should there be the least occasion for this qualifying clause? Was it not practicable to obtain all the facts precisely as they were? There were certainly gentlemen in this city, of trustworthy character, who were competent to give a history perfectly reliable; were they called upon to do so? If not, is there not some ground of complaint, that, in a matter affecting, not alone the validity of the opinion of the editor of the Buffalo Medical Journal, (which is of no great moment,) but the interests of a medical institution, and the character of professional brethren, conclusions and actions should not be based on something more definite than rumors or doubtful reports? We repeat, we do not say but that the gentlemen who signed the letter were fully conversant with all the facts in the matter, but it would seem to be a fair inference from the language quoted, that they were not entirely confident, themselves, on this point, and, if not, why should they not, before deciding on administering a public rebuke, in justice to others, as well as themselves, have taken pains to have obtained complete information, more especially when it was not difficult to have done so?

Entertaining the presumption that some of the signers may have affixed their names under erroneous impressions, we will proceed to give, briefly, a statement of the occurrence, as related to us by individuals who were present, and in whose representations we have implicit confidence. We fully concur with our friends that the subject is not one properly admitting of public discussion, but we do not apprehend that the description which we shall give, will shock the sensibilities of a majority of the patrons of this Journal, and, besides, it is due to those of our readers who do not reside in Buffalo, that in occupying so much space with this topic, we should, at least, give them an opportunity to know what it is which has given rise to the occasion for these remarks. The sum and substance of the matter then, is as follows:

During the session, just closed, of the Medical College of Buffalo, the Professor of Midwifery conceived the plan of illustrating his instructions with the living subject. The plan was submitted to every member of the Medical Faculty of the College, and received the unqualified approbation of each individual member. A female, shortly to be confined, was found, who was willing, after a full representation of the matter, to undergo parturition before the class. With her it was entirely voluntary; for aught we know to the contrary, her character is good; no improper influences were brought to bear on her decision, and she has, since her confinement, made written testimony that she was in every way kindly treated, that she considered herself fortunate in having better accommodations and attendance than she would otherwise have expected, and that she has no cause whatever for complaint or dissatisfaction. In deciding to accept the proposition, she stated, (what we have no reason to think was not true,) that a motive determining her acceptance was derived from the supposed usefulness of the illustration.

The patient remained in the family of the Janitor of the college two weeks before her confinement occurred. During this period, opportunity was afforded to every member of the class, individually, to auscultate for the intra-uterine sounds, but with the same regard to delicacy as in any private case.

When labor occurred, agreeably to arrangement, the graduating class were summoned. The patient remained, as before, in one of the apartments occupied by the family of the Janitor. The class assembled in another part of the building. During the progress of the labor, each member of the class, singly, was permitted to visit the patient, and, in the presence of the Professor, to make a vaginal examination, with the same observances of

...icacy as in any private case. Finally, as the head of the fœtus was about emerging from the *os externum*, all the graduating class were invited to enter the room, and, the Professor supporting the perinæum, the patient lying on a bed on her left side, in the customary mode, attended by the wife of the Janitor, the mechanism of delivery was witnessed, with no exposure beyond what was requisite for the illustration. The class remained in the room somewhat less than half an hour, and during this period no word was uttered, except by the Professor, nor was there, in any mode, the least deviation from that decorum suited to the occasion, and in keeping with the objects for which they were present. During the time that the class remained in the room, the delivery took place, and all the duties subsequent thereto, were performed by the Professor.

To complete the history, it is only necessary to add, that nothing occurred, subsequent to the labor, to affect unfavorably either mother or child.

This is the instance of demonstrative midwifery which occasioned the resolutions and correspondence, published in the last number of our Journal, together with the editorial remarks, which some of our esteemed professional brethren have deemed deserving of a formal protest.

We may claim, as we think, on behalf of all concerned, directly or indirectly, in this matter, good intentions. We will not believe, that any one of the gentlemen whose signatures are attached to the letter, will charge upon the Medical Faculty of the College, a disposition to pander to vulgar curiosity, or to sentiments of a grosser nature. This being assumed, we ask, whence the occasion for a "severe rebuke," whatever may have been the character of the "exhibition"? To merit a severe rebuke, implies something more than a mistake to have been committed; it involves, not an error of judgment alone, but an offence, proceeding from bad designs, or directed to bad objects. If there are no grounds for a harsh judgment of motives in this case, (and this we leave the reader to decide,) then we submit, that a remonstrance would be the most that a self-constituted board of adjudication was called upon to pronounce. This, if dictated in a spirit of kindness, would undoubtedly have been received, not only with respect, but gratitude; but more especially if the remonstrance had been private and confidential, instead of public.

But why is a severe rebuke called for? The communication specifies the reasons, which we will briefly consider, seriatim.

The first reason assigned is, "that it [i. e. the practice rebuked] is wholly unnecessary for the purpose of teaching." We are not aware that its absolute necessity is contended for; the question, rather, is, whether its utility is sufficient to counterbalance the objections which may be made to it. Now that it is useful, seems to us sufficiently clear, without much argument. The mechanism of parturition occupies a considerable share of oral instructions in midwifery. The teacher endeavors to illustrate it by drawings, models, etc. Will it be said that the opportunity to superadd to these means of illustration clinical demonstrations, is of no account to the student? If so, we may ask in reply, why is it desirable to illustrate other subjects, by actual objects? why will not plates, and cuts, answer as well as actual dissections, for instruction in anatomy and pathology! There would be more room for discussion of this point, if the plan of demonstrative midwifery were now, for the first time, submitted for the approbation of the profession. It is not an original or novel undertaking, except in this country. We are credibly informed, that it is pursued by distinguished medical teachers in France and Germany, and, for aught we know, in other countries. Strange that it has not occurred to any members of the medical profession of this country to quote the practice of European institutions as evidence in favor of the higher character of American schools! Strange, too, that, with some little acquaintance with medical literature, we should not have happenened to fall, somewhere, upon an expression of disapprobation of the practice prior to the present instance!

The next reason stated is, that it is "unprofessional in manner." If it be meant by this, that accouchments are not ordinarily conducted, in all respects, as in this instance, the fact will, of course, be conceded. In this instance the professional services rendered were for purposes of instruction, in addition to the usual objects. We have heard no intimation, from any one, that the patient did not receive the best efforts of the professional skill of the professor. Indeed, the circumstances under which the services were rendered, would be expected to secure for the patient his best efforts. We confess we are unable to perceive the force which this reason, as expressed, was intended to convey. Demonstrative midwifery, as a custom in medical practice, is not inculcated, more than that the accoucheur should carry to the bedside of his parturient patients the plates and manikins which had assisted him in qualifying himself for the practical duties of this branch of medical science. Its sole purpose is educational, and it should be, of course, so restricted.

The last reason given is, that it is, "grossly offensive, alike to morality and common decency." We readily admit the full force of such objections provided they exist. If it can be shown to be offensive to morality we stand ready, nay, we shall be rejoiced to acknowledge that we are in error, and to solicit the favorable oversight of the medical profession, not only of Buffalo, but the United States, in having presumed to advocate it. But assertion, however dogmatic, is not proof, and we might leave the subject, by simply asking for some arguments, before consenting to change our views. We will not adopt this course, but the object of the few words we shall say, will simply be to suggest certain trains of reflection, not to discuss the subject. Let the medical reader, then, consider what should be the legitimate moral effect of witnessing a case of parturition, in connection with instructions in midwifery, upon a class of young gentlemen just about to enter upon the responsibilities of medical practice. Will the effect be moral, or immoral? We appeal for a decision of this question to the experience of medical gentlemen. All practitioners have been medical students. We may be in error, but we are free to say, that, in our judgement, if any member of a graduating class could possibly have connected with that event, levity, or any unworthy associations, clinical observation is calculated to correct, rather than encourage them.

We must add, that we deeply regret to see such a possibility suggested in the letter which we have published. We should be sorry if such a supposition were to be deliberately countenanced by medical men. It may be said, the insinuation relates exclusively to ocular demonstration; but, if true as regards the eye, it would be equally so as regards another sense. The complexion of sentiments does not depend upon the avenue through which fostering sensations are received, but upon that principle which perceives and feels—the mind. "To the pure all things are pure." We will say no more on this point. It remains to notice the charge of indecency. It is easy to pronounce this word, but what do we understand by it? It evidently has a relative, not an abstract significancy. Many of the pictures employed for scientific purposes, would become obscene representations, if diverted from their proper purposes. The law punishes immodest exposures, but, in so doing, it takes cognizance of the intention, as well as the act. A transaction is indecent, not from its intrinsic character, but from its tendency and object. We ask the reader simply to apply this distinction to the present case. If he decides that, in view of the end or design, the charge of indecency holds good, we say that, to be consistent, he must contend for a radical reform in the study of anatomy and other branches of medical science. Museums containing certain wax and other preparations imitating nature to the life, should be indicted as nuisances; demonstrations with the subject on the table of the anatomist, before a class of students, should be curtailed within the limits of propriety, and our text books of Physiology, and Obstetrics, should be subjected to a rigidity of expurgation, which would reduce, in no small degree, their bulk, and deprive them of a goodly share of their illustrations.

With these remarks, thrown off in haste, we conclude this article, already too much extended. We have thought it but due to ourselves, in reply to the letter criticising, in terms of severity, an opinion which we had honestly formed, and in all sincerity expressed, to defend that opinion, inasmuch as we are still so unfortunate as to differ from those of our professional brethren who have united in expressing their disapprobation of it. We have endeavored to reply plainly, but with no feelings of irritation. If the tone of our remarks should seem to denote other than the most respectful and kindly feelings, we have done ourselves an injustice which we should regret. We do not profess to hold in very high esteem that pride of consistency which resists conviction; and if we should become satisfied that we are in error, we should deem recantation neither a hardship, nor a reproach. But until we become converted to the sentiments of our friends, who certainly are at liberty to differ from us, we must reserve the privilege both of holding to our opinion, and of endeavoring to sustain it.

